# Vacancy-Mediated Bound Magnetic Polarons as the Driving Mechanism for Ferromagnetism in Fe-Doped SnO_2_ Nanowires

**DOI:** 10.1021/acsomega.5c10549

**Published:** 2026-01-23

**Authors:** David Montalvo, Do Minh Hoat, Virginia Gómez-Vidales, Wencel José de la Cruz Hernández, Santiago Camacho-López, Karime Carrera, Víctor Orozco, Jonathan Guerrero-Sánchez, Manuel Herrera

**Affiliations:** † Centro de Nanociencias y Nanotecnología-Universidad Nacional Autónoma de México Ensenada, Ensenada, Baja California 22800, México; ‡ Institute of Theoretical and Applied Research, 374802 Duy Tan University, Hanoi 100000, Vietnam; § Instituto de Química, Universidad Nacional Autónoma de México, Circuito Exterior s/n, Ciudad Universitaria, Ciudad de México C.P. 04510, México; ∥ Departamento de Óptica, Centro de Investigación Científica y de Educación Superior de Ensenada, Ensenada, Baja California 22800, México; ⊥ 7180 Centro de Investigación en Materiales Avanzados, Chihuahua, Chihuahua C.P. 31136, México; ∞ School of Engineering and Technology, 374802 Duy Tan University, Da Nang 550000, Vietnam

## Abstract

We report that room-temperature ferromagnetism in Fe-doped SnO_2_ nanowires arises from the interaction between Fe^3+^ dopants and singly ionized oxygen vacancies (V_O_′), mediated through bound magnetic polarons (BMPs). Combining experimental characterization with density functional theory (DFT), we demonstrate that although isolated oxygen vacancies are intrinsically nonmagnetic, their presence between Fe atoms stabilizes ferromagnetic coupling through shared BMP electrons. Raman spectroscopy and XPS confirmed the substitutional incorporation of Fe^3+^ into the SnO_2_ lattice, while CL and EPR revealed the presence of oxygen-deficient environments and directly identified the singly ionized oxygen vacancy centers (V_O_′), whose density increases with Fe incorporation. Magnetic measurements showed enhanced saturation magnetization and coercivity, directly correlated with V_O_′ signals. DFT calculations further supported these findings by identifying Fe–V_O_–Fe complexes as the most stable configurations under O-rich conditions. This joint experimental–theoretical study provides microscopic evidence that vacancy–dopant interactions drive ferromagnetism in Fe-doped SnO_2_ nanowires. The results highlight a defect-mediated mechanism that establishes oxide-based dilute magnetic semiconductors as promising candidates for spintronic applications.

## Graphical abstract

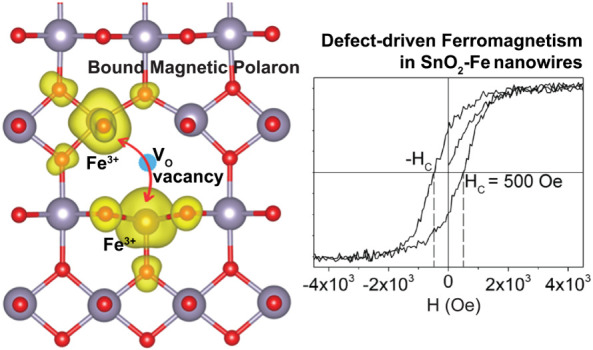

## 1 Introduction

Dilute magnetic semiconductors (DMS) have emerged as a compelling class of materials for next-generation spintronic technologies, owing to their ability to combine charge and spin functionalities within a single platform.^1^ These materials hold great promise for the development of spin-based devices such as spin transistors, magnetic sensors, and nonvolatile memory, where room-temperature spin polarization and control are essential.^2,3^

Among the various oxide-based DMS candidates, tin dioxide (SnO_2_) has attracted particular interest due to its inherent *n-type* conductivity, wide band gap (3.6 eV), chemical stability, and optical transparency. When doped with transition metals such as iron (Fe), SnO_2_ has been reported to exhibit room-temperature ferromagnetism (RTFM).^1,4,5^ Fe presents several advantages as a dopant compared with other transition metals: its ionic radius in octahedral coordination (Fe^3+^: 0.645 Å) is close to that of Sn^4+^ (0.690 Å), enabling substitution at Sn sites with minimal lattice distortion.^6^ In addition, Fe^3+^ carries a relatively large magnetic moment (∼3 μB per ion),^7^ and is abundant and low-cost, making it suitable for large-scale applications. Its abundance and low cost make it ideal for developing technologies in large volumes. Furthermore, doping with Fe maintains the optical transparency of SnO_2_. The coexistence of magnetic order and transparent-conducting behavior makes Fe-doped SnO_2_ a promising functional component for oxide-based spintronic devices, where spin-polarized transport, magnetic layers, and transparent contacts are required. Importantly, Fe incorporation preserves the optical transparency of SnO_2_. The coexistence of magnetic order, transparent conducting behavior, and chemical stability makes Fe-doped SnO_2_ a promising functional material for oxide-based spintronic devices requiring spin-polarized transport, magnetic layers, and transparent contacts.

Despite substantial experimental and theoretical progress, the fundamental origin of ferromagnetism in Fe-doped SnO_2_ remains a subject of debate. Various mechanisms have been proposed, including carrier-mediated exchange interactions (e.g., RKKY), double exchange, and defect-induced magnetism.^8,9^ Among these, the role of point defects, especially oxygen vacancies (V_O_), has gained significant attention as a decisive factor influencing the observed magnetic properties.^10–12^ Factors such as nanostructure size, lattice strain, dopant oxidation state, and defect concentration have all been shown to critically affect magnetic ordering in DMS.^13^

In particular, the Bound Magnetic Polaron (BMP) model has emerged as a promising framework for explaining defect-driven ferromagnetism in oxide semiconductors.^14–16^ According to this model, electrons localized at point defects, such as single-ionized oxygen vacancies (V_O_′), interact with the magnetic moments of nearby Fe^3+^ ions, resulting in the formation of localized ferromagnetic regions (polarons). When these regions overlap sufficiently, they establish long-range ferromagnetic order across the material.^5,17,18^ In this study, we explore the relationship between structural defects and the ferromagnetic behavior of Fe-doped SnO_2_ nanowires synthesized by a physical vapor deposition method. Using a combination of cathodoluminescence (CL) and electron paramagnetic resonance (EPR) spectroscopy, we identify and characterize oxygen vacancies and their role in mediating ferromagnetic interactions. Our findings provide strong experimental support for the BMP model as the primary mechanism behind RTFM in these nanostructures and contribute to a deeper understanding of defect-mediated magnetism in oxide-based DMS materials.

## 2 Materials and Methods

### 2.1 Experimental Section

Undoped SnO_2_ nanostructures were synthesized by thermal evaporation of high-purity SnO_2_ powder (Aldrich Co., 99.999%) in a horizontal quartz-tube furnace (Lindberg/Blue M). Approximately 50 mg of SnO_2_ powder was placed in an alumina boat at the center of the tube. SiO_2_/Si­(100) substrates (Ted Pella, Inc.) were positioned downstream at a distance of 12–15 cm from the source material, where the temperature ranges between 650–750 °C. Before heating, the system was evacuated to 1 × 10^–2^ Torr and subsequently backfilled with ultrahigh-purity argon (Infra Co., 99.999%) to a working pressure of 130 mTorr.^19^ During growth, an Ar flow of 10–20 sccm was maintained using a mass-flow controller. The furnace temperature was then raised to 1300 °C at a ramp rate of 20 °C/min and held for 45 min to promote vapor–solid growth of SnO_2_ nanowires. Fe-doped SnO_2_ nanowires were prepared using the same configuration, except that iron­(II) acetate (C_4_H_6_O_4_Fe, Aldrich Co., 99.999%) was uniformly mixed with the SnO_2_ powder in the source boat at concentrations corresponding to the values listed in Table 1. The presence of the Fe precursor lowers the local oxygen chemical potential at the evaporation zone, necessitating an adjustment in the growth temperature accordingly (650–720 °C at the substrate position). After the dwell time, the furnace was allowed to cool naturally to room temperature under continuous Ar flow to prevent postgrowth oxidation. All samples were stored in sealed containers under ambient conditions prior to characterization.

**Table 1:** Growth Parameters and Elemental Composition of Undoped and Fe-Doped SnO_2_ Nanowires Synthesized at Different Temperatures, Determined by EDS and XPS^a^

		EDS (at. %)			XPS (at. %)		
Sample	Growth Temperature (°C)	Sn ± 0.3	O ± 0.3	Fe ± 0.1	Sn 3p_3/2_ ± 2.9	O 1s ± 7.0	Fe 2p_3/2_ ± 0.2
1	650	33.4	66.6	-	-	-	-
2	720	32.9	66.6	0.5	28.8	69.6	1.6
3	650	32.9	66.4	0.7	28.6	69.5	1.9

a Fe incorporation into the SnO_2_ lattice is confirmed.

The elemental composition of the samples was determined using energy-dispersive X-ray spectroscopy (EDS) equipped with an Oxford X-Max detector. Quantitative analysis was performed using INCA software (Oxford Instruments) following standard calibration procedures. X-ray photoelectron spectroscopy (XPS) measurements were carried out using a SPECS system equipped with a PHOIBOS WAL analyzer and an Al Kα source (1486.6 eV). Peak fitting and quantification were conducted using CasaXPS software (Version 2.3.24PR1.0, 1999–2021, Casa Software Ltd.). Quantitative analysis was based on the relative sensitivity factors (RSF) provided by CasaXPS: Sn 3p_3/2_ = 9.35, O 1s = 2.93, and Fe 2p_3/2_ = 14.8. The typical uncertainty associated with quantitative XPS analysis is 5–10% (relative).^20^ Crystal structure characterization was performed on a Philips X’Pert X-ray diffractometer using Cu–Kα radiation (λ = 0.154 nm). Raman spectra were obtained using a Dimension-P2 λs system with a 532 nm Nd:YAG excitation laser.

X-ray photoelectron spectroscopy (XPS) and Auger electron spectroscopy (AES) analyses were carried out using PHI 535 and SPECS systems, respectively, both equipped with aluminum anodes. High-resolution XPS spectra were recorded using 300 scans and calibrated to the C 1s peak at 284.8 eV. All spectra were deconvoluted using CasaXPS (Version 2.3.24PR1.0, 1999–2021, Casa Software Ltd.).^21^ AES measurements were performed with a 3 keV electron beam as the excitation source. Electron paramagnetic resonance (EPR) spectra were acquired using a JEOL JES-TE300 spectrometer operating in X-band mode with a modulation frequency of 100 kHz and a cylindrical TE_011_ cavity. The external magnetic field was calibrated with a JEOL ES-FC5 gaussmeter, and spectra were recorded in first derivative mode at a microwave frequency of 9.44 GHz and a power of 20 mW. Signal processing and simulations were performed using ES-IPRITS/TE (Data System Version 3) software. Transmission electron microscopy (TEM) and localized EDS analyses on individual nanowires were carried out using a JEOL JEM-2100F (STEM) operating at 200 keV. Morphology characterization was conducted using a JEOL JSM-7800F scanning electron microscope. Cathodoluminescence (CL) measurements were performed at room temperature over the UV–visible spectral range using a Gatan MonoCL4 system coupled to a JEOL FIB-4500 SEM. All spectral deconvolutions, including Gaussian fitting and extraction of fwhm and correlation coefficients (*R* ^2^), were performed using the MagicPlot software (Version 2.9.3). The magnetic behavior of the samples was determined using a vibrating sample magnetometer (VSM) attached to an Evercool-Physical Properties Measurement System from Quantum Design. Magnetization measurements were carried out at room temperature.

It should be noted that techniques such as Raman spectroscopy, CL, TEM, and VSM do not yield statistical data sets for which error bars are typically defined. The structural and spectroscopic features reported in this work were verified by analyzing multiple nanowires in TEM and CL measurements, and by performing repeated measurements on each sample for the VSM technique.

### 2.2 Theory and Calculations

To gain a deeper understanding of the potential formation of the Bound Magnetic Polaron (BMP) and the way it stabilizes the Fe neighbors, we performed density functional theory calculations considering several scenarios. The calculations were carried out in the Vienna Ab initio Simulation Package,^22–24^ which uses the projector-augmented wave method to sample the ion-electron interaction.^25,26^ The electronic states were expanded using plane waves with an energy cutoff of 500 eV. The exchange and correlation term in the Kohn–Sham Hamiltonian was treated according to the PBE parametrization.^27^ The bulk SnO_2_ structure crystallized in the *rutile* structure and space group *P*4_2_/*mnm*. Our optimized lattice parameters are *a* = 4.74 Å and *c* = 3.21 Å, which agree well with the experimental values.^28^ Structural optimization was achieved when the energy differences were lower than 1 × 10^–4^ and the force components were lower than 0.01 eV/Å. The k-point meshes used in this work were selected based on convergence tests performed for both the bulk and defect-containing supercells. For the *rutile* SnO_2_ primitive cell, an 8 × 8 × 12 Monkhorst–Pack grid was found to converge the total energy, forces, and magnetic moments within 1 meV/atom. The defect calculations were performed in a 2 × 2 × 3 supercell, whose enlarged real-space dimensions reduce the size of the Brillouin zone proportionally. Accordingly, a 4 × 4 × 4 mesh provides a k-point density equivalent to the 8 × 8 × 12 grid used in the bulk. This choice ensures fully converged electronic and magnetic properties while maintaining comparable sampling density along each reciprocal-space direction.

## 3 Results and Discussion

Figure 1 displays the X-ray diffraction (XRD) patterns of all samples, exhibiting peaks corresponding to the *rutile*-type tetragonal structure of SnO_2_ (PDF card #88-0287). No additional peaks related to secondary phases, such as iron oxide compounds, were detected. This confirms that Fe atoms were successfully incorporated into the SnO_2_ lattice without the formation of segregated phases. The main diffraction peaks, (110), (101), and (211), agree with previously reported patterns for SnO_2_ nanowires,^29,30^ supporting both the phase purity and crystallographic orientation of the synthesized materials.

**Figure 1:**
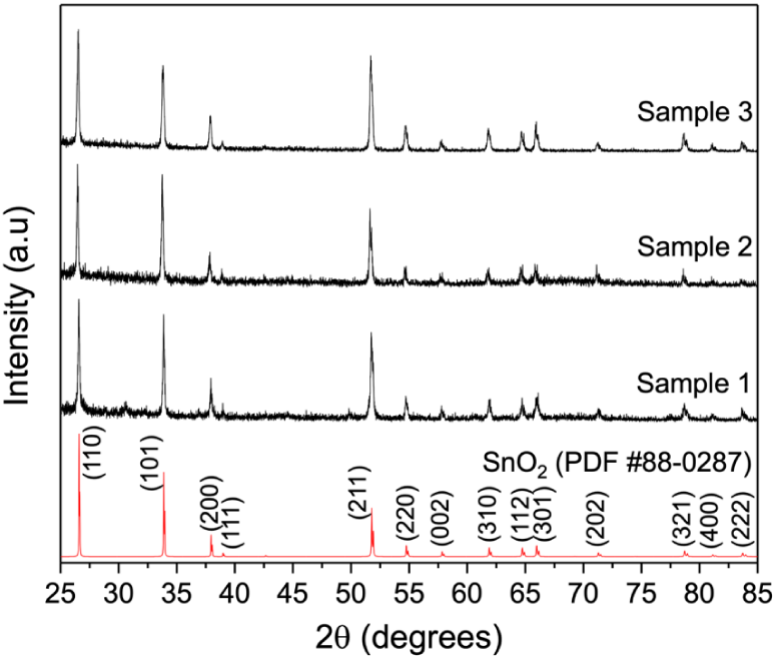
XRD patterns of Samples 1–3, indexed to the *rutile*-type tetragonal SnO_2_ (PDF #88-0287). The absence of secondary phases confirms phase purity, while weak reflections (*) correspond to metallic Sn traces.

To further probe lattice dynamics and assess possible distortions introduced by Fe incorporation, Raman spectroscopy measurements were employed to evaluate the vibrational modes of SnO_2_ nanowires. Tetragonal *rutile* SnO_2_ crystallizes in the *P*4_2_/*mnm* space group (point group D_4_h), for which group-theory predicts four first-order Raman-active optical phonon modes at the Γ point: A_1g_, B_1g_, B_2g_ and E_g_.^31^ In good agreement with previous experimental studies on bulk crystals and nanostructures, the observed Raman peaks at ∼475 cm^–1^, ∼632–634 cm^–1^ and ∼770–774 cm^–1^ were assigned to the E_g_, A_1g_ and B_2g_ modes, respectively.^32^ The A_1g_ mode corresponds to symmetric in-plane stretching of Sn–O bonds, the B_2g_ mode also involves in-plane stretching, although it is typically weaker, and the E_g_ mode is associated with bending of the Sn–O bonds.^33,34^ Figure 2 presents the Raman spectra of the samples. In the undoped SnO_2_, distinct peaks at 474, 632, and 774 cm^–1^ were observed, corresponding to the E_g_, A_1g_, and B_2g_ modes, respectively. In Fe-doped samples, these modes remain clearly visible. The reduced intensity of the E_g_ and B_2g_ modes in Sample 2 is attributed to the lower nanowire density on the substrate, which decreases the effective Raman scattering volume.^35,36^ It should be noted that Raman intensity in nanowire ensembles depends strongly on the nanowire surface density and the available interacting volume, rather than solely on crystallinity. A lower density of nanostructures, therefore, yields weaker Raman signals, without implying diminished crystallinity, as confirmed independently by XRD. The vibrational signatures in Figure 2 confirm that the *rutile* crystal structure is preserved after Fe incorporation, since the Raman-active modes (A_1g_, E_g_, and B_2g_) appear at the same characteristic positions reported for undoped *rutile* SnO_2_.^33,34^ Moreover, no detectable shifts are observed in these modes upon Fe doping. A slight decrease in intensity and a modest broadening of the E_g_ and B_2g_ modes are nevertheless observed in the Fe-doped samples. This behavior is commonly attributed to local lattice distortions arising from Fe^3+^ → Sn^4+^ substitution and to the increased concentration of oxygen-vacancy-related defects, both of which reduce phonon coherence length. The lower intensity observed in Sample 2 is therefore consistent with its reduced nanowire density rather than with any crystalline degradation.

**Figure 2:**
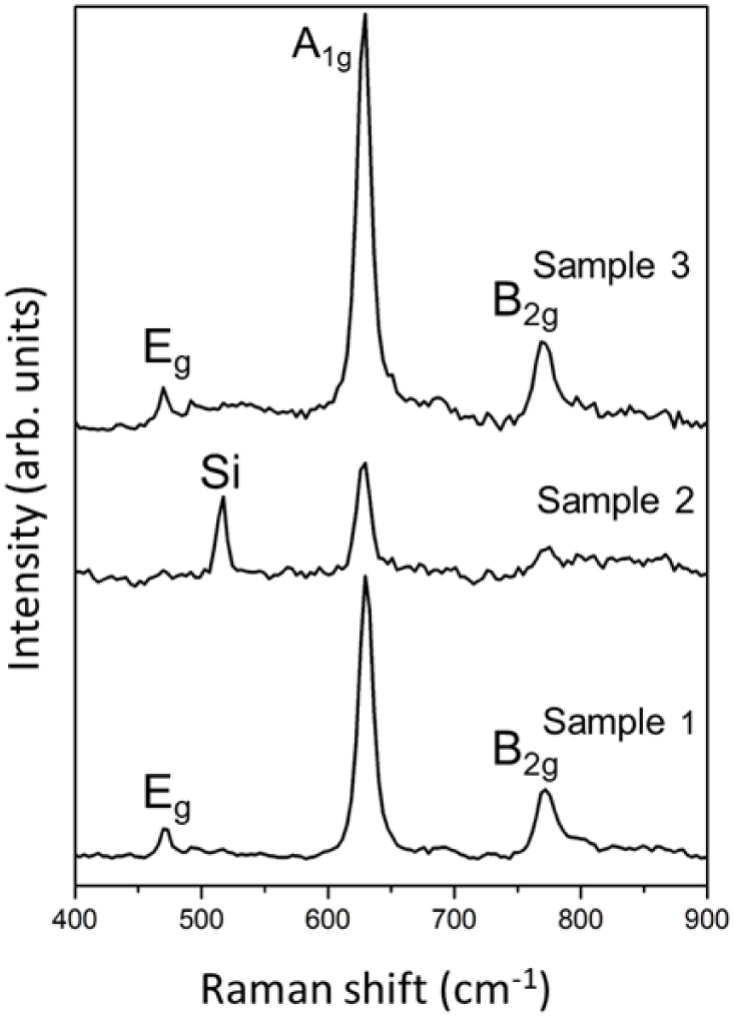
Raman spectra of Samples 1–3, showing the Raman-active E_g_, A_1g_, and B_2g_ modes of *rutile* SnO_2_. A peak from the Si substrate is observed in Sample 2. The slight band broadening in Fe-doped samples indicates lattice distortion induced by Fe substitution.

To determine the structural morphology of the samples, scanning electron microscopy (SEM) was employed. Figure 3 shows SEM images revealing that both undoped and Fe-doped SnO_2_ samples consist of elongated nanowires with similar morphology and uniform distribution across the substrate. The reproducibility of these features among all samples indicates that the growth process is stable and well-controlled. As confirmed by TEM measurements (Figures 4 and 5), both undoped and Fe-doped SnO_2_ nanowires exhibit diameters below 200 nm.

**Figure 3:**
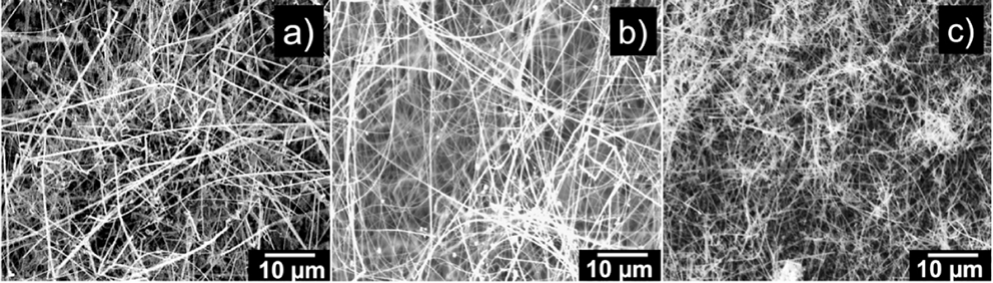
SEM images of (a) undoped SnO_2_ nanowires (Sample 1) and (b, c) Fe-doped SnO_2_ nanowires (Samples 2 and 3). All samples exhibit dense and uniform nanowire networks across the substrate surface.

**Figure 4:**
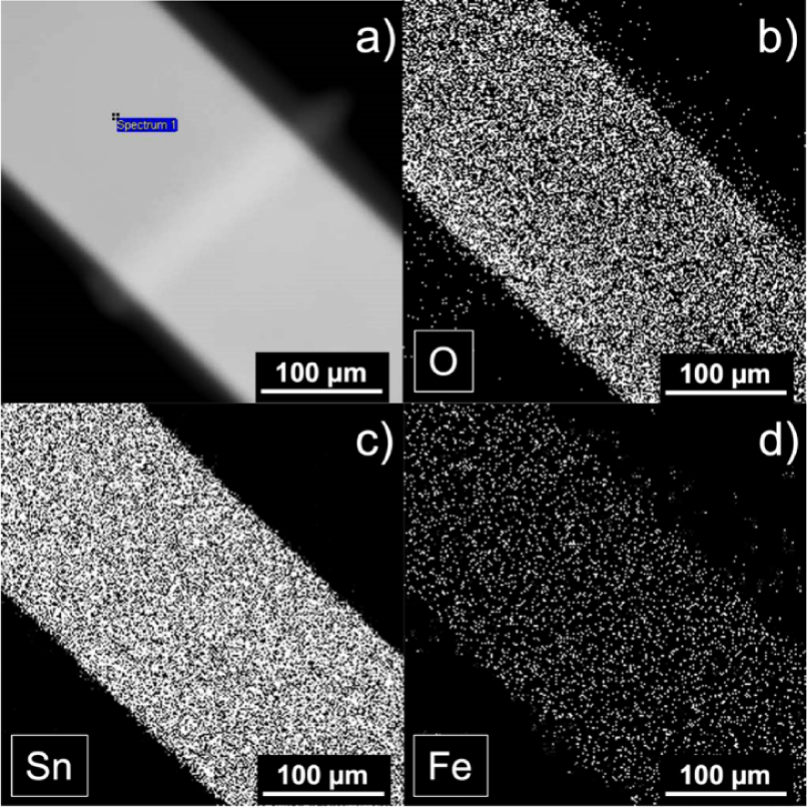
(a) TEM image of a Fe-doped SnO_2_ nanowire (Sample 3) with corresponding EDS elemental maps of (b) O, (c) Sn, and (d) Fe, confirming the homogeneous spatial distribution of dopants.

**Figure 5:**
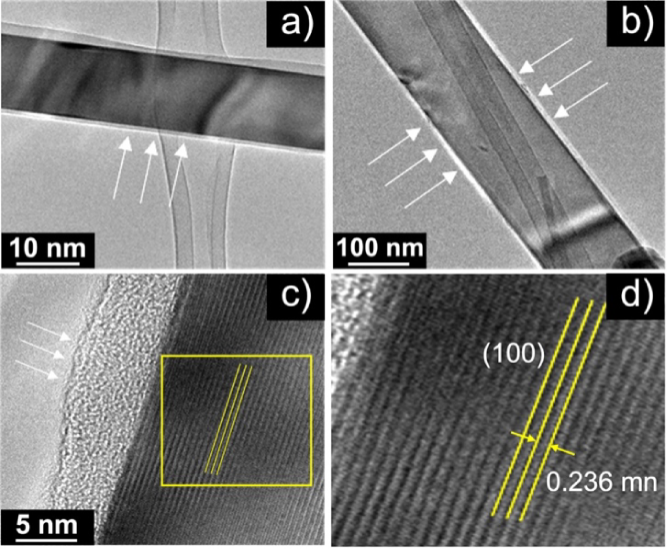
TEM images of Fe-doped SnO_2_ nanowires: (a) Sample 2 and (b) Sample 3, both showing an amorphous carbon surface layer (arrows). (c) Higher magnification of Sample 2, revealing a 2–5 nm amorphous carbon coating. (d) HR-TEM close-up of Sample 2, showing high crystallinity with well-resolved (100) lattice fringes (0.236 nm).

To complete the morphological observations, elemental mapping of individual SnO_2_ nanowires was performed. Figure 4a presents a TEM micrograph of a Fe-doped SnO_2_ nanowire (Sample 3), along with EDS elemental distribution maps for oxygen (O), tin (Sn), and iron (Fe) [Figure 4b–d]. The O and Sn maps confirm the expected uniform distribution of the host lattice elements. The Fe map shows a continuous and homogeneous distribution of dopants across the entire nanowire, with no detectable Fe-rich agglomerates or secondary phases. This nanoscale chemical uniformity supports the substitutional incorporation of Fe into the SnO_2_ lattice. Although the Fe concentration in Sample 3 is only 0.7 at%, the EDS maps in Figure 4d clearly show a uniform distribution of Fe along the nanowire.^19,37^ Previous studies have shown that Fe exhibits high solubility in *rutile* SnO_2_, with homogeneous substitutional incorporation reported up to at least 10–15 at%. In contrast, the present work intentionally employs much lower Fe concentrations, since our objective is to synthesize diluted magnetic semiconductors (DMSs) based on SnO_2_, where the dopant level must remain within the dilute regime and well below the solubility limit in order to avoid the formation of secondary magnetic phases.

To further elucidate the structural quality at the nanoscale, transmission electron microscopy (TEM) analysis was performed. TEM images of Fe-doped SnO_2_ nanowires (Sample 2) are shown in Figure 5a–d. An amorphous surface layer was identified in the HRTEM images by the absence of lattice fringes at the nanowire edges, in contrast to the crystalline core, where the SnO_2_ lattice planes are clearly resolved. The thickness of this layer (∼5 nm) was measured directly from the calibrated HRTEM micrographs. Enhanced carbon signals observed in the Auger spectra, together with the use of iron­(II) acetate as a precursor, indicate that this amorphous shell corresponds to carbon-rich residues formed during precursor decomposition. A close-up [Figure 5d] reveals a lattice spacing of 2.3 Å, matching the (100) interplanar distance of *rutile* SnO_2_.

Figure 6 shows HR-TEM images of Fe-doped nanowires from Samples 2 and 3. Sample 2 exhibits growth along the [010] direction, with lattice spacing of 1.6 Å and 2.3 Å corresponding to the (220) and (100) planes, respectively.^38^ Sample 3 exhibits growth along the [100] direction, with interplanar spacings of 2.3 Å and 3.4 Å corresponding to the (100) and (110) planes, respectively. These growth directions are consistent with previous reports, such as the study by Leonardy et al., which used HRTEM and SAED analyses to demonstrate that SnO_2_ nanowires frequently elongate along the [100] axis under thermal evaporation growth conditions.

**Figure 6:**
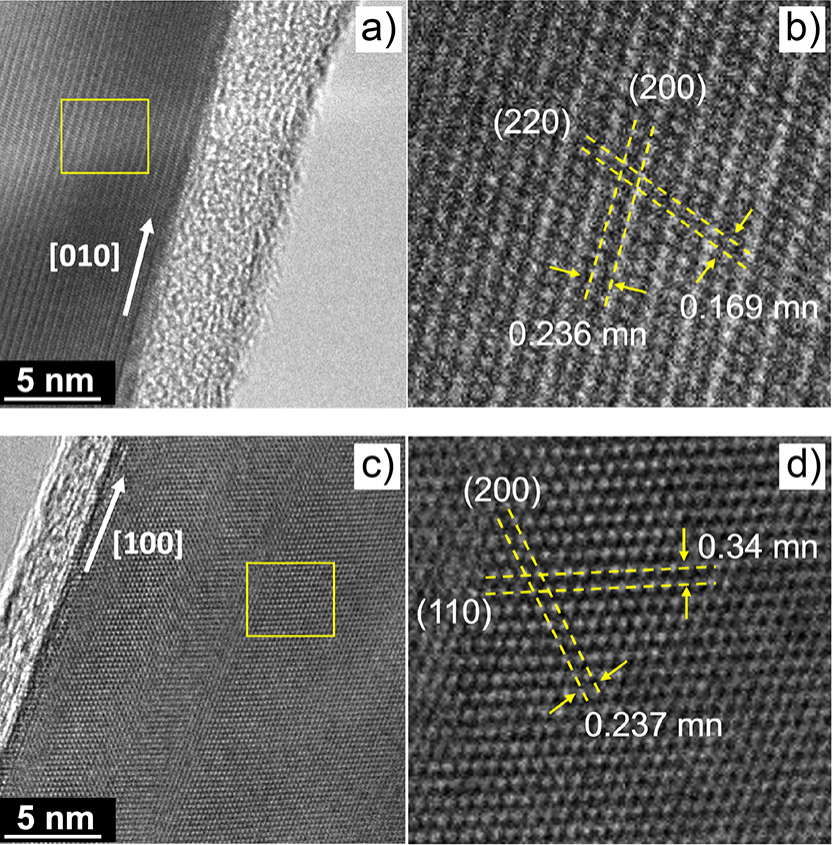
HR-TEM images of Fe-doped SnO_2_ nanowires: (a, b) Sample 2 and (c, d) Sample 3. Growth directions along [010] (Sample 2) and [100] (Sample 3) were identified, with corresponding interplanar spacings indicated in the images.

These observations are also consistent with a previous report of Herrera et al. (2013), who studied Mn-doped SnO_2_ nanowires synthesized by thermal evaporation. Their HRTEM and SAED analyses identified [100] as one of the predominant growth directions, in agreement with our results.^39,40^

While TEM provides direct evidence of lattice order and growth direction, complementary spectroscopy techniques are required to assess surface composition and chemical states. Figure 7a–c presents Auger spectra. The characteristic Sn (MNN) and O (KVV) peaks, centered at 421, 429, and 510 eV,^41^ respectively, appear in all samples. A peak at 274 eV becomes more pronounced in Fe-doped samples, indicating increased carbon presence,^42^ consistent with TEM results showing residual from Fe precursor decomposition.

**Figure 7:**
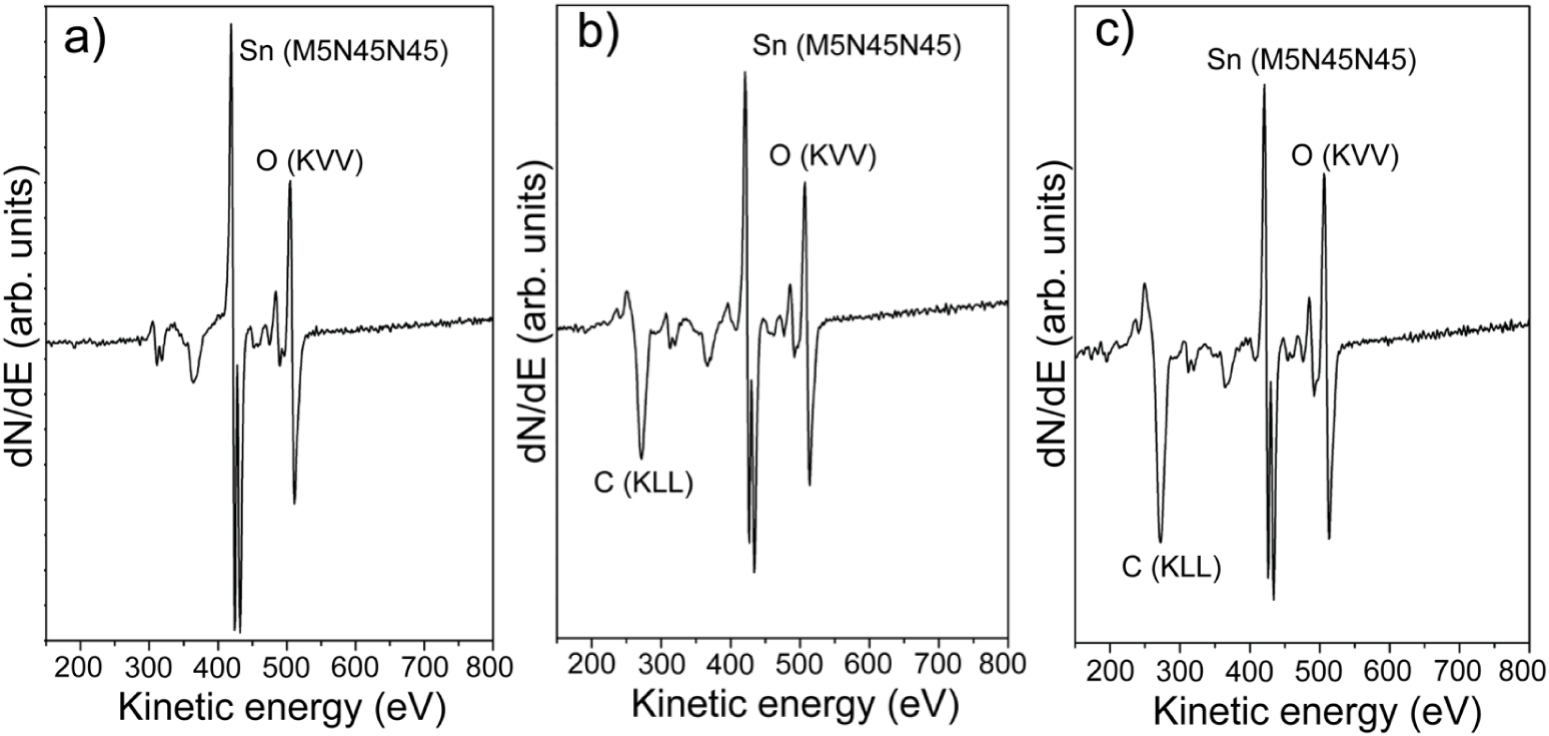
Derivative Auger spectra of (a) Sample 1, (b) Sample 2, and (c) Sample 3, showing characteristic Sn (M_5_N_45_N_45_) and O (KVV) features. Fe-doped samples exhibit increased C (KLL) signals attributed to precursor decomposition.

X-ray photoelectron spectroscopy (XPS) was employed to investigate the atomic composition and the chemical state of elements present on the surface of the SnO_2_ nanowires. It should be noted that the Sn/O ratios determined by XPS and EDS are not expected to coincide (Table 1), because XPS probes only the outer ∼5 nm of the nanowire surface, whereas EDS reflects the composition of the entire nanowire volume. As shown in the HRTEM images (Figure 5), the nanowires possess a ∼5 nm amorphous, oxygen-deficient surface layer. This shell has a strong influence on the XPS signal but a negligible effect on EDS, leading to the observed difference in apparent stoichiometry.

Figure 8 shows the Sn 3d core-level spectra, with two distinct peaks centered at 486.3 eV (3d_5/2_) and 494.7 eV (3d_3/2_), corresponding to a spin–orbit splitting of 8.4 eV. This value is consistent with the expected splitting for Sn^4+^ ions bonded to O^2–^ in the SnO_2_ lattice.^43^ Figure 9a presents the deconvoluted O 1s core-level spectrum of the undoped SnO_2_ sample, which reveals three components centered at 530.8, 532.2, and 533.8 eV. The peak at 530.8 eV is attributed to lattice oxygen (Sn–O bonds), while the peaks at 532.2 and 533.8 eV are associated with absorbed hydrocarbon species,^31,44^ and physisorbed water molecules, respectively. Figure 9b,c display the O 1s spectra for the Fe-doped samples (Samples 2 and 3), which exhibit the same three components. However, a noticeable increase in the 532.2 eV component is observed, indicating a higher presence of surface carbon species, originated by the decomposition of the acetate-based Fe precursor. Figure 10a,b present the Sn 3p_3/2_ core-level XPS spectra for Samples 2 and 3, displaying a main peak centered at 716.6 eV. This peak partially overlaps with the Fe 2p_3/2_ signal located at 711.5 eV, which is characteristic of Fe^3+^ ions.^45^ The presence of this peak confirms the incorporation of iron in the +3 oxidation state, in agreement with previous reports on Fe-doped SnO_2_ nanostructures.^46,47^ However, due to the very low Fe content in our samples (0.3–0.7 at%), the Fe 2p region does not exhibit a sufficiently strong or well-resolved signal to enable reliable quantitative analysis, an expected limitation in dilute transition-metal-doped oxides. For this reason, Electron Paramagnetic Resonance (EPR) measurements were additionally performed to unambiguously verify the presence of Fe^3+^ ions.

**Figure 8:**
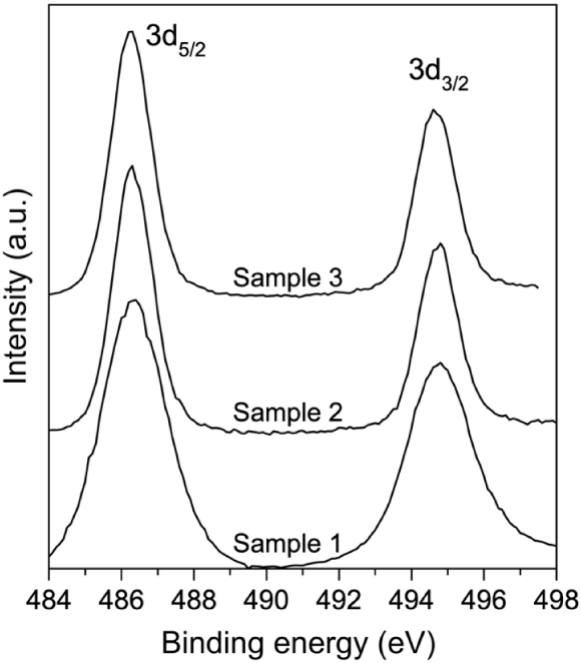
High-resolution XPS core-level spectra of Sn 3d for Samples 1–3, showing the spin–orbit doublets 3d_3/2_ characteristic of Sn^4+^, confirming the *rutile* SnO_2_ phase.

**Figure 9:**
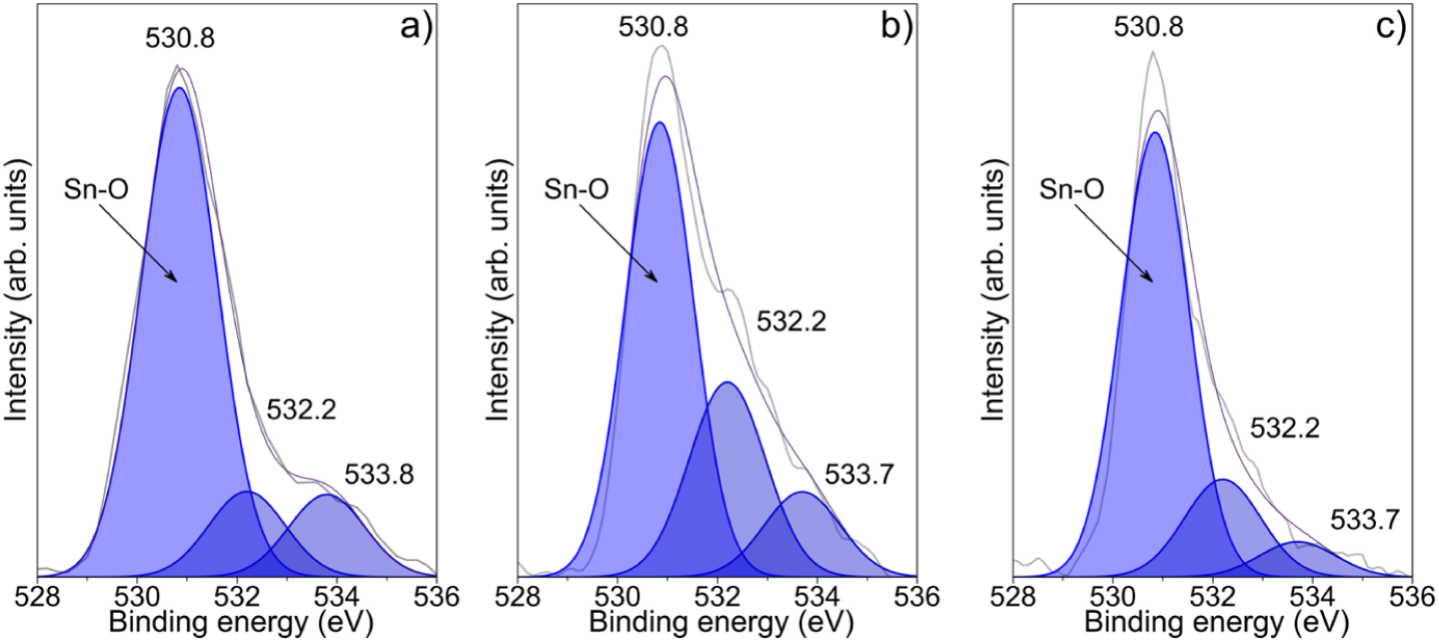
Deconvoluted O 1s XPS core-level spectra of (a) Sample 1, (b) Sample 2, and (c) Sample 3 with peaks attributed to lattice oxygen (∼530.8 eV), adsorbed hydrocarbons (∼532.2 eV), and physisorbed water (∼533.7 eV).

**Figure 10:**
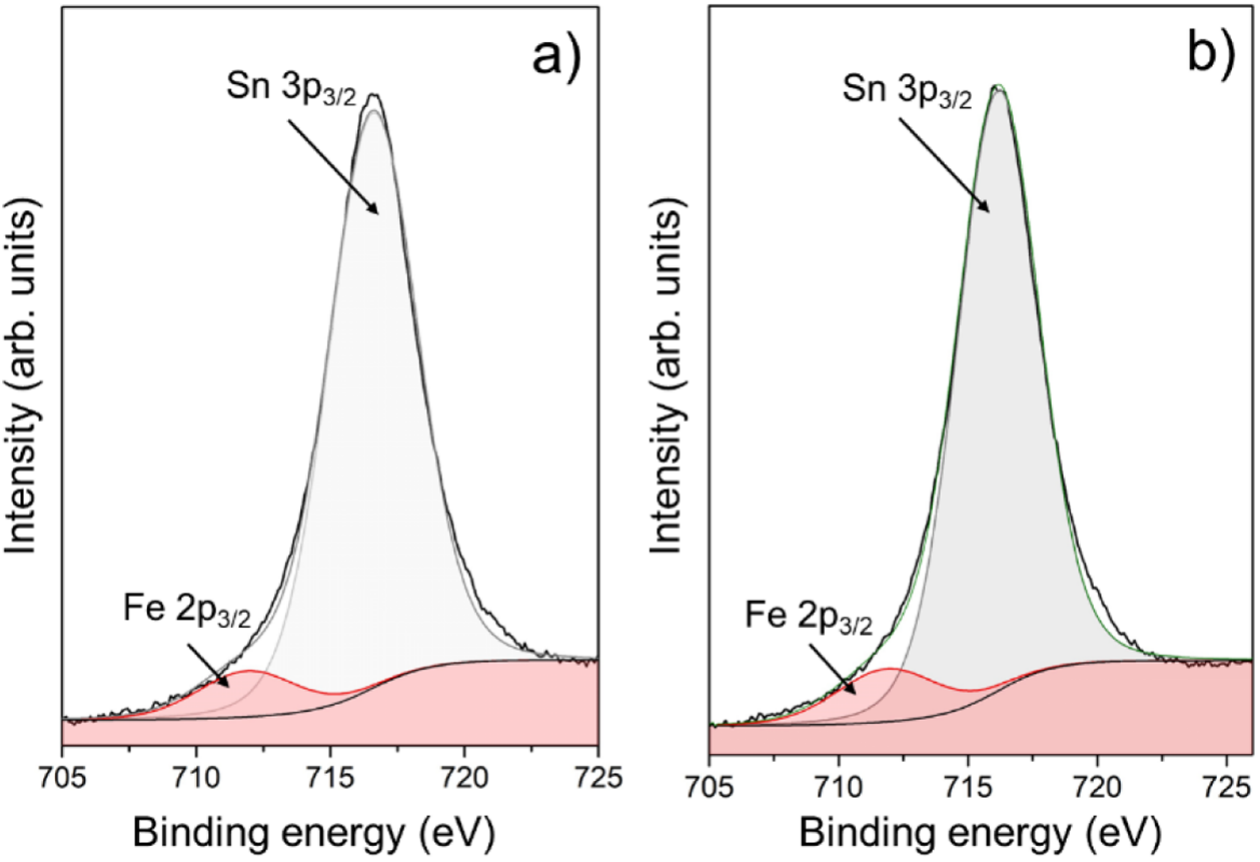
XPS spectra of Sn 3p_3/2_ overlapping with Fe 2p_3/2_ for (a) Sample 2 and (b) Sample 3. Deconvolution confirms the presence of Fe^3+^ ions substituting Sn^4+^ lattice sites.

Since XPS revealed the incorporation of Fe^3+^ ions and surface oxygen species, electron paramagnetic resonance (EPR) measurements were carried out to probe paramagnetic centers and point defects, providing complementary information.^48^ ^,^ EPR experimental spectra were analyzed and simulated using the EasySpin toolbox in MATLAB (see Supporting Information), which allowed us to extract the *g*-tensor values corresponding to distinct paramagnetic species. Figure 11a presents EPR spectrum of the undoped SnO_2_ sample (Sample 1), recorded in the magnetic field range of 100–600 mT (curve 1), along with its corresponding simulated spectrum (curve 2). The signal exhibits a *g*-value of 2.17 and a peak-to-peak width (Δ*H*_p–p_) of 210 mT, consistent with an *S* = 1/2 system attributed to superoxides $\left(O_{2}^{-}\right)$ species adsorbed on the SnO_2_ surface.^49,50^ Figure 11b shows the EPR spectrum of Sample 3 measured over the same field range. The experimental signal (curve 1) exhibits a broad with Δ*H*_p–p_ of 230 mT, and the simulated spectrum (curve 2) yields a *g*-value of 2.07. This value is characteristic of Fe^3+^ ions incorporated into the SnO_2_ lattice,^48^ ^,^ and is consistent with the Fe^3+^ oxidation state identified in the XPS spectra of Figure 10.

**Figure 11:**
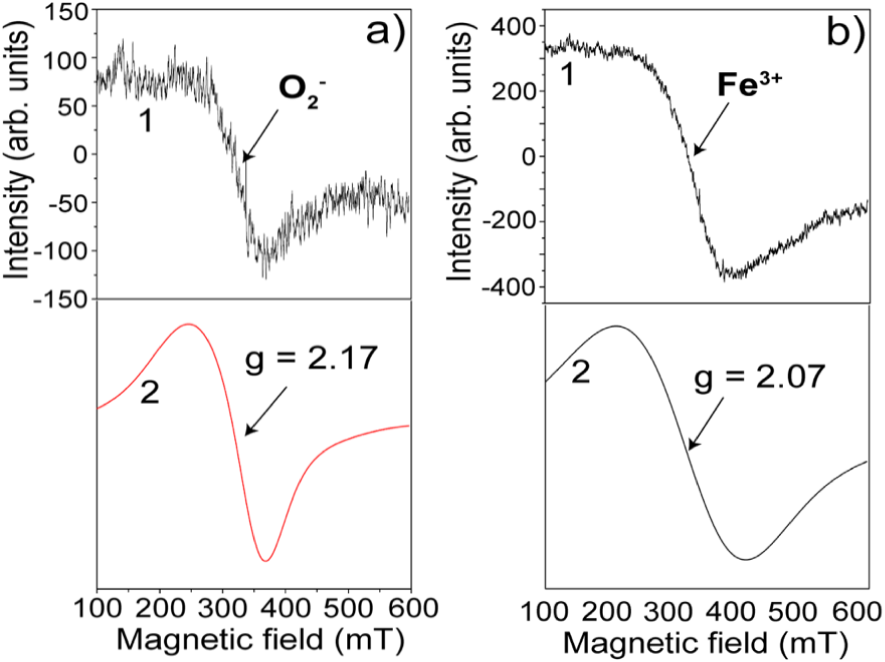
EPR spectra of (a) Sample 1 and (b) Sample 3, showing signals from O_2_^–^ (*g* = 2.17 ± 0.0003) and Fe^3+^ (*g* = 2.07 ± 0.0003), respectively. Curve 1 corresponds to the experimental spectrum and curve 2 to the simulation.

Figure 12a,b focus on the narrow spectral region between 335 and 340 mT, where high-resolution EPR scans were obtained from Samples 1 and 3, respectively. Both samples show a sharp signal with Δ*H*_p–p_ of 0.35 mT (curve 1), accurately reproduced by simulations (curve 2). The extracted *g*-values are 2.0031 for Sample 1 and 2.0029 for Sample 3. Considering the manufacturer-specified measurement uncertainty (±0.0003), both signals correspond to the same *S* = 1/2 transition associated with singly ionized oxygen vacancies $\left(V_{O}^{\prime }\right)$ . These values are in excellent agreement with those previously reported for centers in SnO_2_. For example, Popescu et al. (2001) reported a *g* = 2.003 for defects in SnO_2_ nanostructures, and similar *g-*values have been observed in *rutile*-type oxides such as TiO_2_, further supporting this assignment.^51–53^

**Figure 12:**
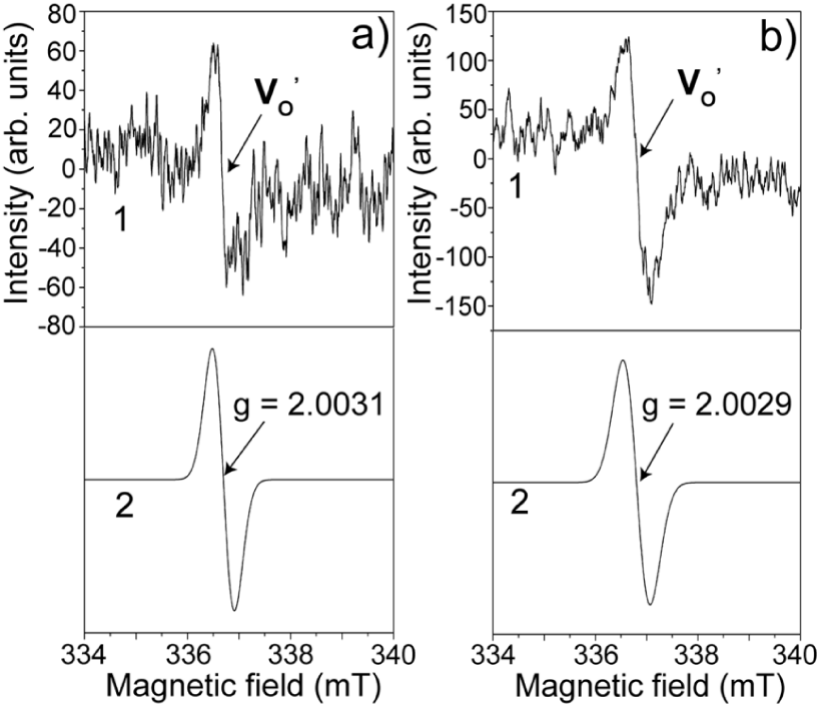
High-resolution EPR spectra showing signals of singly ionized oxygen vacancies (V_O_′) in (a) Sample 1 and (b) Sample 3. Curve 1 corresponds to the experimental spectrum and curve 2 to the simulation, with *g*-values of 2.0031 ± 0.0003 and 2.0029 ± 0.0003, corresponding, characteristic of V_O_′.

The defect states identified by EPR motivated a detailed optical investigation through cathodoluminescence (CL), which is highly sensitive to oxygen-vacancy-related emissions. Figure 13a displays the CL spectrum of the undoped SnO_2_ sample, showing a broad emission band that was deconvoluted into three Gaussian components centered at 2.07, 2.50, and 2.80 eV. The fitting analysis, performed using MagicPlot (version 2.9.3), yielded an excellent correlation coefficient (*R* ^2^ = 0.9985), with full width at half-maximum (fwhm) values of approximately 0.51 eV for each Gaussian component. The emission centered at 2.07 eV has been consistently reported in the literature; for example, Maestre et al. (2004) studied the cathodoluminescence of SnO_2_ nanospheres annealed under different temperatures and oxygen atmospheres and identified this feature, commonly referred to as the “orange band”, as originating from oxygen-vacancy-related defect states. Their findings support our assignment of the 2.07 eV component to defect centers involving oxygen vacancies.^39,54^ First-principles calculations have shown that this emission is specifically associated with surface V_O_ defects located on bridging oxygen atoms that are 6-fold coordinated to Sn atoms.^55,56^ The 2.50 eV is generally ascribed to in-plane oxygen vacancies at the SnO_2_ surface, particularly involving oxygen atoms coordinated at distorted angles around 130°.^55^ Additionally, this green emission has been linked to electronic transitions between shallow donor levels and surface V_O_ states.^57,58^ The 2.80 eV component corresponds to the blue luminescence, which is typically observed in the 2.70–2.80 eV range. This emission is attributed to a combination of structural defects,^59^ oxygen-related lattice distortions,^60^ and the presence of surface V_O_ centers.^55,61,62^ When examining the effect of Fe incorporation, CL spectra of Fe-doped SnO_2_ samples [Figure 13b,c] revealed the same type of emission of V_O_-related defects are present regardless of doping. However, variations in the relative intensities of these emissions were observed with increasing Fe content. Overall, the CL results suggest that Fe doping does not introduce additional types of luminescent centers, but it does influence the density of existing oxygen vacancy defects involved in the optical emissions. This observation is consistent with the EPR analysis, which also indicated the generation of $V_{O}^{\prime }$ defects induced by Fe^3+^ incorporation into the SnO_2_ lattice.

**Figure 13:**
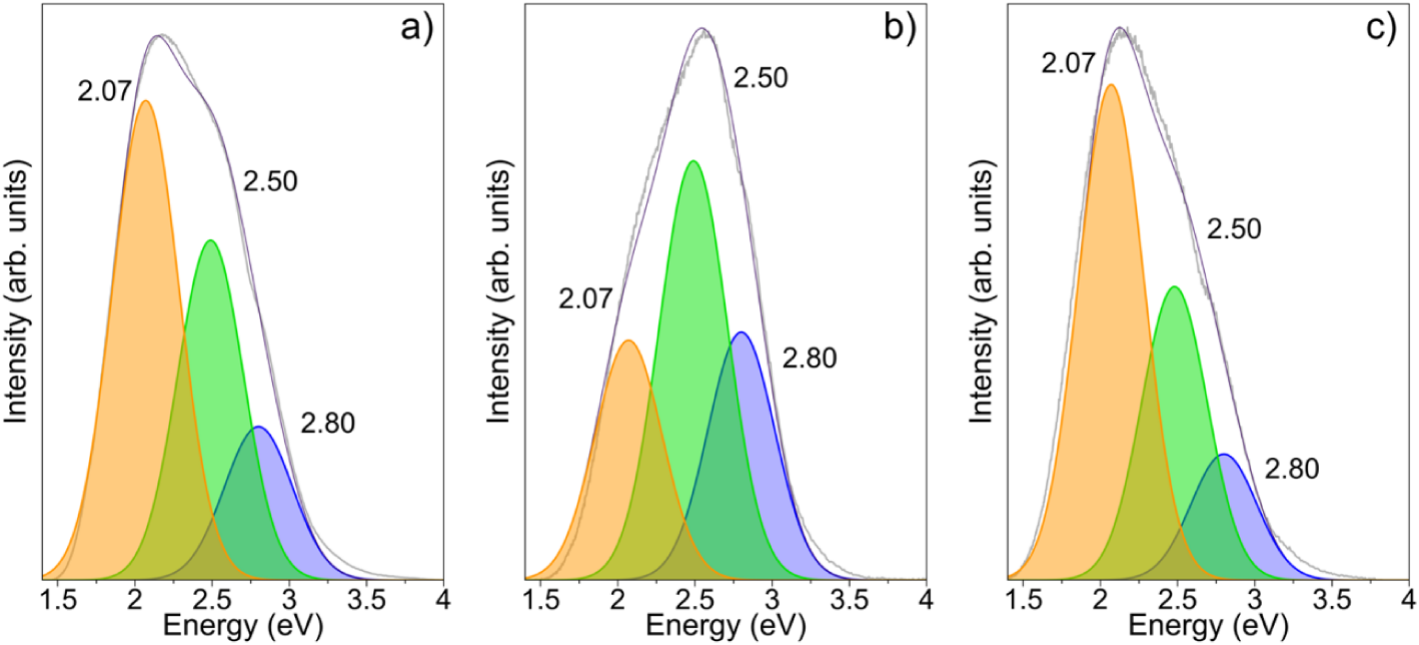
Cathodoluminescence spectra of (a) Sample 1, (b) Sample 2, and (c) Sample 3, showing emission bands at 2.07, 2.50, and 2.80 eV, which are attributed to distinct oxygen vacancy states and other structural defects.

Given the presence of V_O_′ defects revealed by CL and EPR, magnetic measurements were performed to investigate their possible contribution to the magnetic behavior of SnO_2_ and their interaction with Fe dopants. Figure 14a shows the magnetization versus magnetic field (*M*–*H*) curve of undoped SnO_2_ nanowires (Sample 1) after subtracting the diamagnetic contribution of the Si substrate. To isolate the magnetic signal of the nanowires, the diamagnetic contribution of the Si substrate was removed by fitting the linear high-field portion of the raw *M*–*H* data with a straight-line baseline using MagicPlot (version 2.9.3). This fitted diamagnetic background was then subtracted from the total magnetization, yielding the curves shown in Figure 14.^10^ The sample exhibits clear ferromagnetic (FM) behavior, with a saturation magnetization (*M*_S_) of approximately ±6 × 10^–4^ emu/g and a coercive field (*H*_C_) of 90 Oe. These values are consistent with those reported by Zhang et al. and Montalvo et al. for undoped SnO_2_ nanowires.^63,64^ Previous studies have suggested that the intrinsic FM in undoped SnO_2_ arises from exchange interactions between unpaired electron spins associated with oxygen vacancies,^10,65^ which we confirmed by EPR measurements as single-ionized oxygen vacancies (V_O_′) [Figure 11a]. Upon Fe doping, a clear enhancement of ferromagnetism is observed. The *M*–*H* curve for Fe-doped SnO_2_ nanowires containing 0.5 at% Fe (Sample 2) also displays ferromagnetic behavior, with enhanced *M*_S_ = ±1 × 10^–3^ emu/g and *H*_C_ = 400 Oe [Figure 14b], significantly higher than in the undoped Sample 1. This enhancement is attributed to the incorporation of Fe^3+^ ions in the SnO_2_ *rutile* structure, which promotes the formation of paramagnetic V_O_′ defects. Similar increases in magnetic saturation due to doping with magnetic impurities have been reported in other dilute magnetic semiconductors (DMS).^66–68^ In our case, the formation of V_O_′ defects likely result from a charge imbalance induced by the substitution of Sn^4+^ by Fe^3+^, as supported by the EPR signal in Sample 2 [Figure 11b], which is consistent with our previous finding in SnO_2_:Cr nanowires.^63^ This process can be described using the Kröger–Vink notation^69,70^ as follows:

1 $${\mathrm{Sn}}_{\mathrm{Sn}}^{\times }+V_{O}^{\times }\overset{{\mathrm{Fe}}^{3+}}{\to }{[{\mathrm{Fe}}^{3+}]}_{\mathrm{Sn}}^{\times }+V_{O}^{\prime }$$

where × ,′, and • represent neutral, negatively, and positively charged defects, respectively.

**Figure 14:**
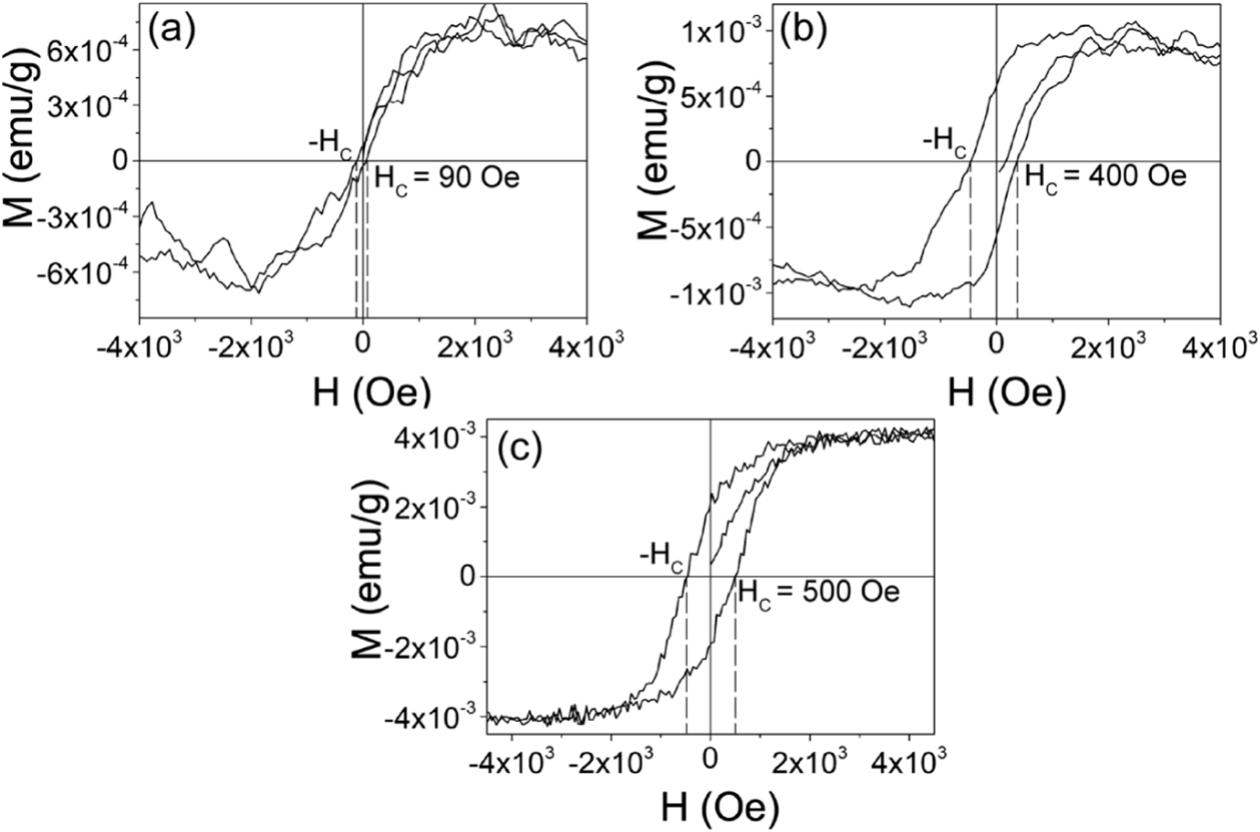
Magnetization–field (*M*–*H*) curves of (a) Sample 1, (b) Sample 2, and (c) Sample 3 after subtracting the diamagnetic contribution of the Si substrate.

Building upon these results, the ferromagnetic behavior can be rationalized within the framework of the Bound Magnetic Polaron (BMP) model.^17^ In this model, the magnetic moment of the spin 1/2 system (V_O_′) couples with that of neighboring Fe^3+^ ions, forming localized magnetic regions called magnetic polarons. The overlap of these polarons facilitates long-range magnetic exchange interactions across the semiconductor lattice. Figure 14c shows the *M*–*H* curve for Sample 3 (0.72 at% Fe), which exhibits further enhanced magnetic properties, with *H*_C_ = 500 Oe and *M*_s_ = ± 4 × 10^–3^ emu/g. This enhancement correlates with the increased EPR signal of V_O_′ defects in Sample 3, suggesting that these spin-1/2 oxygen vacancies play a central role in the FM behavior of SnO_2_:Fe nanowires, in agreement with the BMP mechanism. The higher coercivity in Sample 3 is attributed to the formation of Fe–V_O_ complexes and BMPs, which act as magnetic pinning centers and increase the local anisotropy. This defect-mediated mechanism, widely reported in transition-metal-doped SnO_2_ and other DMS oxides, enhances the resistance to magnetization reversal and explains the larger coercive field observed in the doped sample compared to the undoped sample. We have ruled out the possibility that the observed ferromagnetic response originates from iron-oxide secondary phases, including those present at very low concentrations typically detectable only by Mössbauer spectroscopy. Instead, the combined evidence from Raman, XRD, HRTEM, EDS mapping, XPS, and EPR unambiguously indicates that Fe is incorporated substitutionally into the SnO_2_ lattice. The slight negative slope of *M*(*H*) at high magnetic fields observed in Samples 1 and 2 originates from the strongly diamagnetic Si/SiO_2_ substrate, whose background contribution dominates when the magnetic moment of the nanowires is extremely small. This behavior is typical for oxide nanostructures measured on diamagnetic substrates. Importantly, no evidence of diamagnetic or nonmagnetic impurity phases is found in XRD, Raman, HRTEM, EDS, XPS, or EPR. The ferromagnetic response of the Fe-doped nanowires is therefore intrinsic and arises from defect-mediated mechanisms involving Fe–V_O_ complexes and BMP formation.

To further validate the BMP mechanism, we performed DFT calculations by modeling Fe dopants in SnO_2_ with and without oxygen vacancies (V_O_). Figure 15 shows the atomistic models of Fe-doped SnO_2_ incorporating V_O_. All configurations were generated from a 2 × 2 × 3 supercell of *rutile* SnO_2_ using the Vienna Ab initio Simulation Package (VASP). Fe dopants were introduced by substituting Sn atoms, and oxygen vacancies were created by removing selected O atoms from the supercell. All structures were fully relaxed prior to visualization. The atomic renderings were produced using the VESTA software based on the relaxed VASP coordinates. Fe dopants were incorporated by substituting Sn atoms, and oxygen vacancies were introduced by removing selected O atoms. All structures were fully relaxed before visualization. The figures were rendered using the VESTA software based on the relaxed atomic coordinates. Four configurations were considered within a 2 × 2 × 3 supercell: (i) a pristine SnO_2_ system containing an isolated oxygen vacancy (V_O_) [Figure 15a], (ii) Fe substitution at Sn sites in the vacancy free lattice (Fe-inc) [Figure 15b], (iii) Fe substitution at Sn sites located near an oxygen vacancy (Fe-inc, V_O_ near) [Figure 15c], and (iv) Fe incorporation far from the vacancy (Fe-inc, V_O_ far) [Figure 15d]. These models allowed us to systematically assess how the relative position of Fe and oxygen vacancies influences defect stability and magnetic interactions in the lattice.

**Figure 15:**
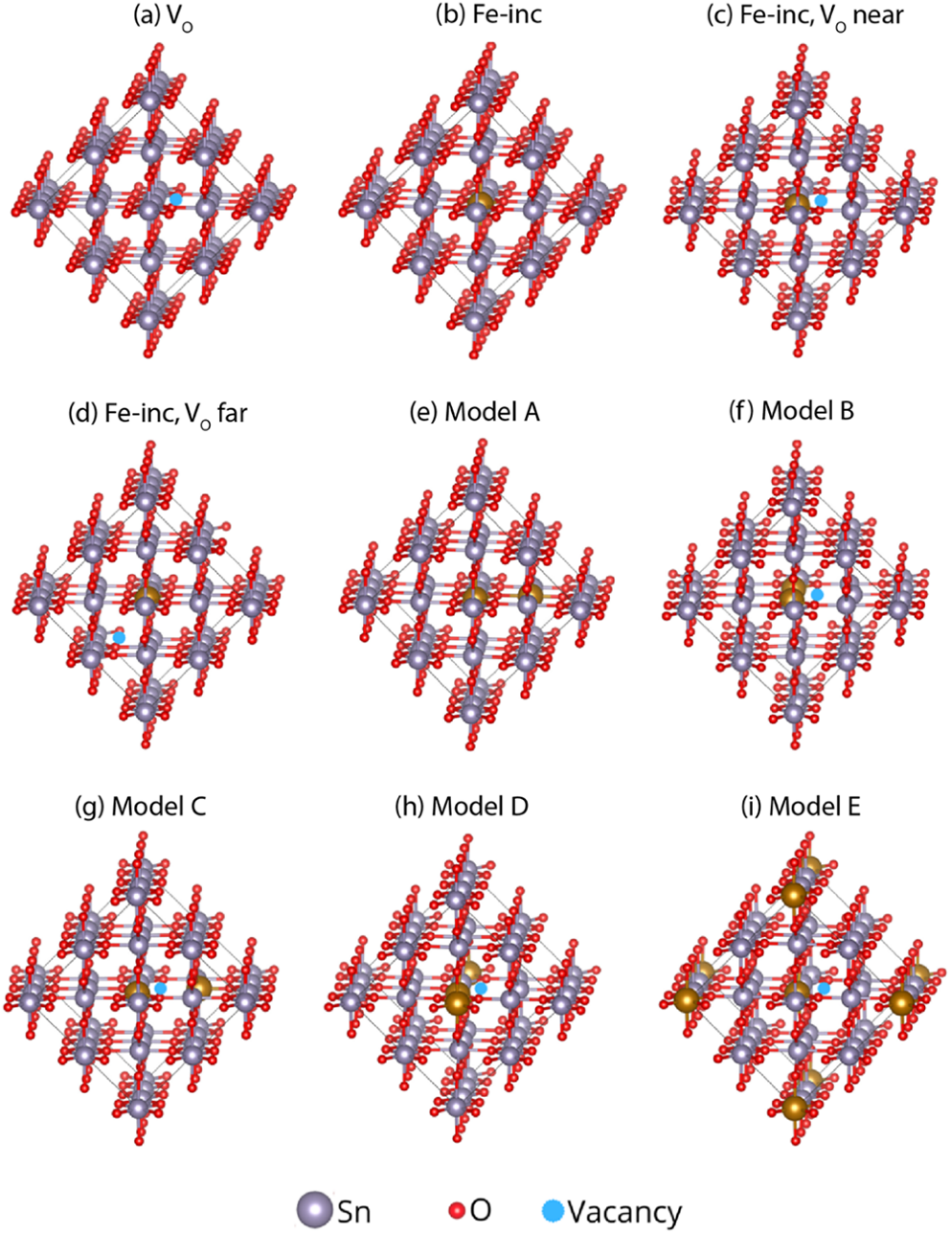
Atomistic models of Fe-doped SnO_2_ incorporating oxygen vacancies (V_O_). Different Fe–V_O_–Fe configurations were simulated to evaluate their relative stability and magnetic interactions. (a) Isolated oxygen vacancy (V_O_), (b) single Fe incorporation (Fe-inc), (c) Fe-inc with V_O_ nearby, (d) Fe-inc with V_O_ farther away, (e) Model A: two Fe atoms substituting Sn without vacancies, (f) Model B: two Fe atoms near a vacancy but interacting through an oxygen atom, (g) Model C: two Fe atoms separated by an oxygen vacancy, (h) Model D: one Fe atom adjacent to the vacancy and the other at a farther site, (i) Model E: one Fe atom adjacent to the vacancy and the other at a distant lattice site. Color code: Sn (violet), O (red), V_O_ (blue).

Guided by our experimental evidence, we further investigate whether oxygen vacancies mediate Fe–Fe interactions in SnO_2_. To this end, we modeled several configurations incorporating two Fe atoms. Model A, two Fe are connected through a bridging O atom [Figure 15e]. Model B corresponds to the same Fe–O–Fe linkage but with a nearby oxygen vacancy, a geometry expected to promote superexchange interactions [Figure 15f]. In Model C, two Fe atoms are directly separated by an oxygen vacancy [Figure 15g]. Model D represents a variation, with one Fe atom located near the vacancy and the second positioned adjacent to the first Fe but farther from the vacancy, enabling us to evaluate whether this arrangement enhances stability [Figure 15h]. Finally, Model E places one Fe atom adjacent to the vacancy and the second at a more distant site within the supercell [Figure 15i].

For the models described above, the total energy cannot be directly used as a reliable criterion for determining the most stable configurations, since the number of atoms differs among them. Instead, we employed the defect formation energy, a well-established stability formalism that is independent of system size and depends only on the chemical potentials of the involved species. To apply this approach, we assume thermal equilibrium between the SnO_2_ bulk and an external reservoir with which atoms can be exchanged. The growth limits were defined by the formation enthalpy of SnO_2_, $\Delta H_{f}^{SnO_{2}}=-1.67\;eV/\mathrm{atom}$ . Under Sn-rich conditions, the chemical potential of Sn is $\mu_{Sn}=\mu_{Sn}^{bulk}$ , whereas under Sn-poor conditions it is given by $\mu_{Sn}=\mu_{Sn}^{bulk}+\Delta H_{f}^{SnO_{2}}$ . Throughout the analysis, the reference zero of energy corresponds to the pristine SnO_2_ bulk structure.

The defect formation energy trends (Figure 16) reveal three dominant stability regimes as the tin chemical potential shifts from Sn-rich (O-poor) to Sn-poor (O-rich) conditions. Under Sn-rich conditions, the isolated oxygen vacancy (V_O_) is the most stable configuration, with a formation energy as low as −0.26 eV. At intermediate conditions, Fe incorporation into the pristine lattice (Fe-inc) becomes the most favorable defect. Finally, under O-rich and extremely O-rich conditions, Model C, consisting of two Fe atoms separated by an oxygen vacancy, emerges as the most stable configuration. Other configurations, such as Model A (two Fe atoms linked by an oxygen atom without vacancies), and Model B (two Fe atoms near a vacancy but interacting via an oxygen atom), are only slightly less stable. This suggests that, under realistic experimental growth conditions, both V_O_ defects and Fe incorporation are expected to coexist, with oxygen vacancies playing a decisive role in stabilizing Fe–Fe interactions. Among the alternative arrangements, Model D (one Fe atom near the vacancy and the other farther away) is marginally more favorable than Model B, underscoring the importance of vacancy placement in defect stability. Although Model E (one Fe adjacent to the vacancy and the other at a distant site) may form due to its relatively low formation energy, it is less likely. Overall, these results highlight that under O-rich conditions the dominant defect complex is Model C, providing strong theoretical support for our experimental evidence that vacancy-mediated ferromagnetism in Fe-doped SnO_2_ nanowires originates from bound magnetic polaron formation.

**Figure 16:**
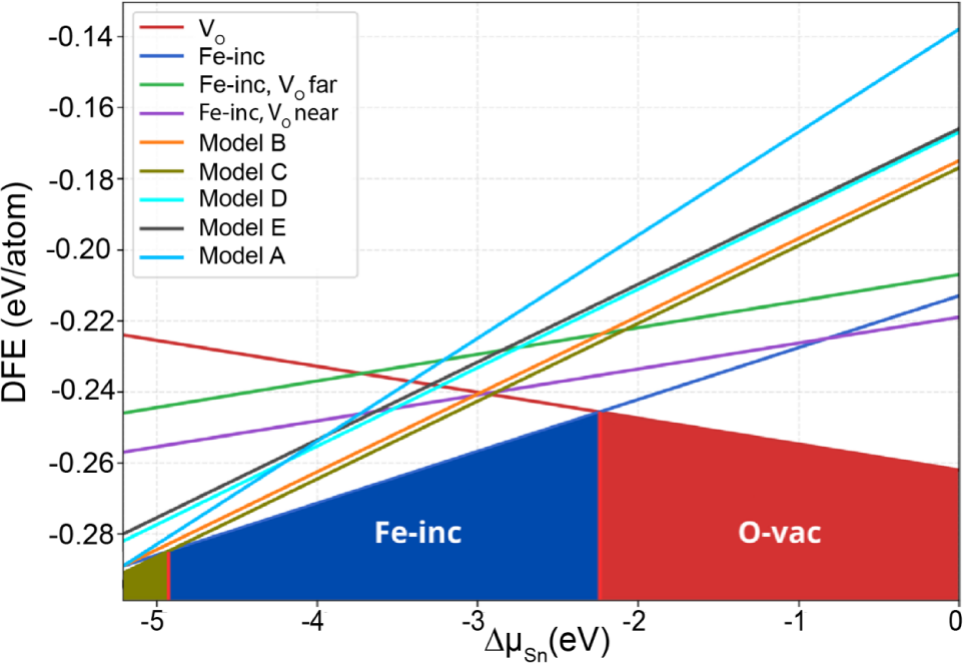
Defect formation energies (DFE) of oxygen vacancy complexes in Fe-doped SnO_2_ as a function of the tin chemical potential $\left(\Delta \mu_{Sn}=\mu_{Sn}-\mu_{Sn}^{bulk}\right)$ . Configurations include isolated oxygen vacancies (V_O_), single Fe incorporation (Fe-inc), and different Fe–V_O_–Fe complexes (Models A–E). Results indicate that Fe–V_O_–Fe complexes are energetically most stable under O-rich conditions, supporting their role in mediating ferromagnetic interactions.

To confirm that ferromagnetism is indeed enhanced through the BMP mechanism, namely, Fe–V_O_–Fe interactions, we analyze the magnetic characteristics of the most stable models. Within the level of approximation used, no intrinsic magnetic character was detected for isolated oxygen vacancies, consistent with the results of Rahman et al.^71^ These authors demonstrated that oxygen vacancies do not directly induce magnetism, since the removal of a neutral oxygen atom leads to the reduction of Sn^4+^ to Sn^2+^. In this scenario, no dangling bonds remain because Sn maintains full coordination with the surrounding oxygen atoms, resulting in nonmagnetic insulating behavior.^71^ Our stability analysis further supports this conclusion: while oxygen vacancies do not themselves polarize, they play a critical role in modulating the magnetic properties of nearby dopants.

Figure 17 presents the spin density isosurfaces for three representative configurations: (a) Fe incorporation in the pristine lattice (Fe-inc), (b) two Fe atoms near a vacancy but interacting through an oxygen atom (Model B), and (c) two Fe atoms separated by a vacancy (Model C). In Figure 17a, magnetism originates mainly from the incorporated Fe atom, which couples ferromagnetically with the neighboring O atoms. The calculated magnetic moments are 4.3 μ_B_ for Fe and 0.12 μ_B_ for O. In contrast, Model B exhibits a clear antiferromagnetic alignment, which is 81 meV more stable than the ferromagnetic ordering. Here, both interactions coexist: antiferromagnetism through p–d superexchange (Fe–O–Fe), and ferromagnetism mediated by the vacancy acting as a spin-polarized ligand. The inset in Figure 17b (isovalue 0.001 e/Å^3^) reveals lobes with opposite spin polarization at the bridging O atom, confirming p–d superexchange between half-filled Fe-3d orbitals mediated by O-2p states. The corresponding magnetic moments are approximately 4.2/–4.2 μ_B_ for Fe and 0.1 μ_B_ for O. The most compelling evidence of vacancy-driven ferromagnetism appears in Model C [Figure 17c], where Fe–V_O_–Fe interactions dominate. In this case, the direct Fe–O–Fe antiferromagnetism pathway is suppressed, and the energy difference between AFM and FM alignments is only 1.8 meV, well below the thermal energy at room temperature (∼25 meV). Thus, thermal fluctuations favor ferromagnetic ordering, especially in the presence of the vacancy-bound single electron (V_O_′) observed experimentally. The spin density isosurface shows polarized lobes spanning the vacancy and both Fe atoms, consistent with a shared BMP electron mediating the ferromagnetic coupling. These results demonstrate that while Fe–O–Fe interactions are primarily antiferromagnetic through superexchange, the introduction of an oxygen vacancy between Fe dopants weakens this interaction and promotes BMP-driven ferromagnetism. This finding aligns with our experimental observations and with previous reports showing that single-ionized vacancies, with a localized electron, facilitate ferromagnetic coupling between dopants.^72^

**Figure 17:**
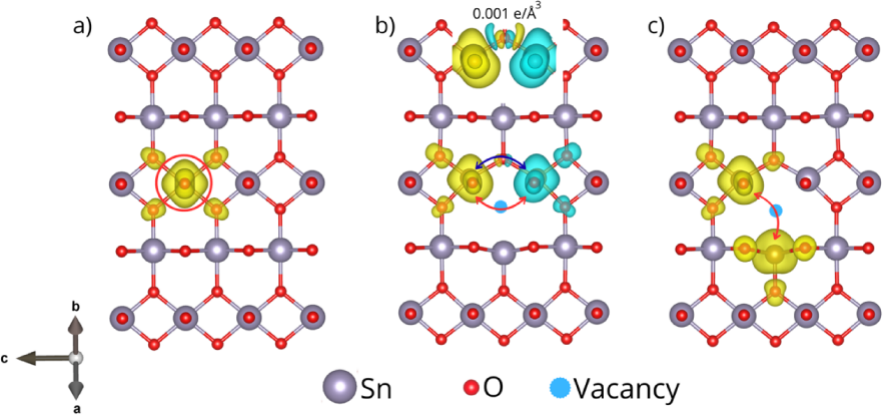
Spin density isosurfaces of (a) isolated Fe incorporation, (b) Fe–O–Fe linkage with an oxygen vacancy, and (c) Fe–V_O_–Fe complex in SnO_2_. Red and blue regions represent ferro-and antiferromagnetic alignments, while yellow and blue lobes indicate spin-up and down spin polarization, respectively. The isosurface level is set at 0.001 e/Å^3^. BMP-mediated coupling is clearly evidenced in the Fe–V_O_–Fe configuration.

In conclusion, our results highlight the fundamental role of oxygen vacancy engineering and controlled transition-metal doping in modifying the magnetic properties of SnO_2_ nanostructures. This study not only elucidates the microscopic origin of ferromagnetism in Fe-doped SnO_2_ nanowires but also provides a framework for tailoring magnetic functionality in oxide-based spintronic devices.

## 4 Conclusions

In this work, we combined structural, spectroscopic, magnetic, and theoretical analyses to unravel the microscopic origin of room-temperature ferromagnetism in Fe-doped SnO_2_ nanowires synthesized by thermal evaporation. A comprehensive set of characterizations (XRD, Raman, TEM, EDS, XPS, and AES) confirmed the substitutional incorporation of Fe^3+^ into the *rutile* SnO_2_ lattice without the formation of secondary phases. EPR and cathodoluminescence measurements consistently identified single-ionized oxygen vacancies (V_O_′) as the dominant point defects, whose density scales with Fe concentration. Magnetic measurements revealed a direct correlation between the density of V_O_′ centers and the systematic enhancement of saturation magnetization and coercivity. DFT calculations provided microscopic validation by demonstrating that isolated oxygen vacancies are intrinsically nonmagnetic but stabilize ferromagnetic coupling when bridging Fe atoms, forming Fe–V_O_–Fe complexes. Spin-density isosurfaces revealed that the vacancy donates a localized electron, enabling the formation of bound magnetic polarons (BMPs). Theoretical stability analysis further identified Fe–V_O_–Fe complexes as the most favorable defect configurations under O-rich conditions, in excellent agreement with experimental results. Altogether, these findings establish defect–dopant interactions mediated by V_O_′ centers as the driving mechanism for ferromagnetism in Fe-doped SnO_2_ nanowires. Beyond clarifying a long-standing debate, this study provides a framework for tailoring magnetic functionalities in oxide-based dilute magnetic semiconductors, opening avenues for the rational design of nanostructured materials in spintronic applications.

## Supplementary Material



## Data Availability

The data supporting the findings of this study are available from the corresponding author upon reasonable request. The dataset includes raw structural and magnetic files that are part of ongoing research and, therefore, cannot be made publicly available at this time.

## Notes

MATLAB code used for simulating and fitting the experimental EPR spectra (PDF)
