# Supplementary material for: Vacancy-Mediated Bound Magnetic Polarons as the Driving Mechanism for Ferromagnetism in Fe-Doped SnO2 Nanowires
Source: ACS Omega. 2026 Jan 23;11(5):8141–53. doi: 10.1021/acsomega.5c10549 (PMC12903008; doi:10.1021/acsomega.5c10549)
Supplement: Supplementary file 1 [file ao5c10549_si_001.pdf]

Supporting Information

# Vacancy-Mediated Bound Magnetic Polarons as the Driving Mechanism for Ferromagnetism in Fe-Doped $SnO_2$ Nanowires

David Montalvo[a], Do Minh Hoat[b], Virginia Gómez-Vidales[c], Wencel José de la Cruz Hernández, Santiago Camacho-López[d], Karime Carrera[e], Víctor Orozco[e], Jonathan Guerrero-Sánchez[a] and Manuel Herrera[a*]

[a] Centro de Nanociencias y Nanotecnología-Universidad Nacional Autónoma de México Ensenada, 22800-Baja California, México.
[b] Institute of Theoretical and Applied Research, Duy Tan University, Ha Noi 100000, Viet Nam
[c] Instituto de Química, Universidad Nacional Autónoma de México, Circuito Exterior s/n, Ciudad Universitaria, C.P. 04510, Ciudad de México, México
[d] Departamento de Óptica, Centro de Investigación Científica y de Educación Superior de Ensenada, 22800-Ensenada, Baja California, México.
[e] Centro de Investigación en Materiales Avanzados, Chihuahua, Chihuahua, C.P. 31136, México.

* Corresponding author. Email: zaldivar@cnyn.unam.mx.

**Code S1.** MATLAB code used for the simulation and fitting of the experimental EPR spectrum of the $O_2^-$ signal (g = 2.170)

```
% Importing data
[B,spc] = textread('SnO2 79 596.txt','%f %f');
plot(B,spc);

% Experiment parameters
Exp.mwFreq = 9.44; %GHz
Exp.Range = [min(B) max(B)]; %mT
Exp.nPoints = numel (B);
Exp.Temperature = 298; % Temperature in Kelvin
Exp.CrystalSimmetry = 'P42/mnm'; % Spce Group

% Defining spin systems

Sys1.g =2.17;
Sys1.lwpp = 210;       % O2- radicals
Sys1.weigth = 0.5;

Sys2.g =[2.54,1.95,2.3];
Sys2.gStrain = [2.2,0.8,2];   % sample holder
Sys2.weigth = [0.1,0.3,0.1];

% Next, we specify which parameter we want to be fitted and by how much
% the fitting algorithm can vary it.

Vary1.g =.001;
Vary1.lwpp = 50;
Vary1.weigth =10;

Vary2.g =[0.0001,0.0001,0.0001];
Vary2.gStrain =[0.05,0.05,0.05];
Vary2.weigth = [0.1,0.1,0.1];

% Last-Square fitting
SimOpt.Method = 'perturb';
FitOpt.Method = 'simplex int'; % simplex algorithm, integrals of spectra
esfit('pepper',spc,{Sys1,Sys2},{Vary1,Vary2},Exp,SimOpt,FitOpt);
```

**Code S2.** MATLAB code used for the simulation and fitting of the experimental EPR spectrum corresponding to the $Fe^{3+}$ signal (g = 2.070).

```
% Importing data
[B,spc] = textread('SnO2 Fe M2 79 596.txt','%f %f');
plot(B,spc);

% Experiment parameters
Exp.mwFreq = 9.44; %GHz
Exp.Range = [min(B) max(B)]; %mT
Exp.nPoints = numel (B);
Exp.Temperature = 298; % Temperature in Kelvin
Exp.CrystalSimmetry = 'P42/mnm'; % Spce Group

% Defining spin systems
Sys1.g =2.07;
Sys1.lwpp = 280;       % Fe 3+
Sys1.weigth = 2;

Sys3.g =[2.54,1.95,2.3];
Sys3.gStrain = [2.2,0.8,2];   % sample holder
Sys3.weigth = [0.1,0.3,0.1];

% Next, we specify which parameter we want to be fitted and by how much
% the fitting algorithm can vary it.
Vary1.g =.01;
Vary1.lwpp = 10;
Vary1.weigth =10;


Vary3.g =[0.0001,0.0001,0.0001];
Vary3.gStrain =[0.05,0.05,0.05];
Vary3.weigth = [0.1,0.1,0.1];

% Last-Square fitting
SimOpt.Method = 'perturb';
FitOpt.Method = 'simplex int'; % simplex algorithm, integrals of spectra
esfit('pepper',spc,{Sys1,Sys2},{Vary1,Vary2},Exp,SimOpt,FitOpt);
```

**Code S3.** MATLAB code used for the simulation and fitting of the experimental EPR spectrum corresponding to singly ionized oxygen vacancies ($V_O'$) (g = 2.003).

.

```
% Importing data
[B,spc] = textread('SnO2 Fe M2 329 344.txt','%f %f');
plot(B,spc);

% Experiment parameters
Exp.mwFreq = 9.44; %GHz
Exp.Range = [min(B) max(B)]; %mT
Exp.nPoints = numel (B);
Exp.Temperature = 298; % Temperature in Kelvin
Exp.CrystalSimmetry = 'P42/mnm'; % Spce Group

% Defining spin system
Sys1.g =2.0032;
Sys1.lwpp = .42;      % (Vo')
Sys1.weigth = 1;

% Next, we specify which parameter we want to be fitted and by how much
% the fitting algorithm can vary it.

Vary1.g =.001;
Vary1.lwpp =.01;
Vary1.weigth = .1;


% Last-Square fitting
SimOpt.Method = 'perturb';
FitOpt.Method = 'simplex int'; % simplex algorithm, integrals of spectra
esfit('pepper',spc,{Sys1},{Vary1},Exp,SimOpt,FitOpt);
```
